# Dose-Dependent Dual Effects of Gradient Ionizing Radiation on Neurocognition

**DOI:** 10.3390/ijms27041842

**Published:** 2026-02-14

**Authors:** Xiaokun Jian, Beier Jiang, Sixu Li, Tianjiao Min, Yingwei Xu, Ruoshui Xu, Lina Liu, Ying He

**Affiliations:** 1 Navy Medical Centre, Naval Medical University, Shanghai 200433, China; 2 College of Food Science and Technology, Shanghai Ocean University, Shanghai 201306, China

**Keywords:** ionizing radiation, neurocognition, adaptive response, neuroprotection

## Abstract

Ionizing radiation (IR) exerts complex, dose-dependent biphasic effects on the central nervous system (CNS). This review systematically elucidates the mechanisms underlying the impact of high- and low-dose radiation on neurocognitive function. High-dose radiation (HDR) triggers severe DNA damage, oxidative stress, and neuroinflammatory cascades, leading to neuronal dysfunction, suppression of neurogenesis, and failure of neural circuit reorganization, ultimately resulting in persistent cognitive decline. In contrast, low-dose radiation (LDR) exhibits a unique dual nature: within certain thresholds, it can activate endogenous protective pathways—including DNA repair and antioxidant defenses—thereby promoting neural plasticity and network homeostasis and demonstrating adaptive responses and neuroprotective potential. The research paradigm is shifting from the traditional linear no-threshold (LNT) model towards a dynamic homeostasis model. Future research should prioritize the development of neuroprotective strategies during radiotherapy for high-dose exposure, optimize irradiation modalities, and develop novel radioprotective agents to improve patient outcomes. For LDR, it is crucial to delineate its biological effects and explore its potential for intervening in neurodegenerative diseases. This review aims to provide an integrated theoretical framework for understanding the dose-dependent biphasic regulation of radiation on neurocognition and to outline future directions for developing related protective and therapeutic strategies.

## 1. Introduction

Since the mid-20th century, catastrophic nuclear events—exemplified by the Hiroshima bombing [1] and the Chernobyl disaster [2]—have dramatically increased public awareness of the profound and long-lasting physiological impacts of ionizing radiation (IR) exposure. These catastrophic events highlighted the potential for radioactive materials to cause widespread biological damage, not only through direct exposure but also via secondary routes, including inhalation and ingestion of radioactive isotopes. While environmental radiation exposure remains a significant concern, humans are also exposed to IR in medical, occupational, and industrial settings. These exposures occur in both acute high-dose scenarios, such as radiotherapy and diagnostic computed tomography (CT) scans [3], and chronic low-dose scenarios, such as those encountered in nuclear energy production, aerospace activities, and radiation-related industries [4]. As the medical application of IR has expanded significantly, particularly in the diagnosis and treatment of various diseases, a systematic assessment of its biological effects—particularly on the central nervous system (CNS)—under both high- and low-dose exposure conditions has become a critical and urgent scientific priority.

Ionizing radiation encompasses a wide range of radiation types, including α and β particles, X-rays, γ-rays, protons, neutrons, and heavy ions. These radiation types differ substantially in their linear energy transfer (LET), penetration capability, and radiation quality factors, all of which critically influence their biological effects. Consequently, radiation-induced biological responses are determined not only by the absorbed dose but also by LET, penetration properties, and radiation quality. It is important to note that even at equivalent absorbed doses, high-LET radiation (such as neutrons and heavy ions) generally induces more severe biological damage than low-LET radiation (such as X-rays and γ-rays) [5,6].

According to the United Nations Scientific Committee on the Effects of Atomic Radiation (UNSCEAR, 2008), IR exposure is commonly classified by dose, with high-dose radiation (HDR) defined as acute exposure exceeding 1–2 Gy, primarily associated with radiotherapy, and low-dose radiation (LDR) defined as doses below 100 mGy (0.1 Gy), typically encountered in diagnostic imaging and environmental exposure [7,8]. This review adopts these dose-based classifications when discussing the neurocognitive effects of both high- and low-dose IR.

Traditionally, the CNS was thought to be relatively resistant to radiation-induced damage due to its low cellular turnover and largely post-mitotic nature, leading to the assumption that it was less susceptible to radiation effects than rapidly proliferating tissues. Yet, emerging evidence from epidemiological studies, animal models, and molecular research indicates that the CNS is, in fact, highly sensitive to IR, particularly with respect to cognitive function [9,10,11]. These findings challenge the traditional view of the CNS as largely radioresistant and emphasize the importance of understanding its response to radiation across different doses. While cognitive impairments resulting from HDR, such as those observed in clinical radiotherapy, have been well documented [12,13,14], the effects of LDR on the CNS remain controversial and under active investigation [15]. Some studies suggest that LDR may induce mild neuroinflammation [16], alter synaptic plasticity [17], and lead to cognitive decline [18]. However, other studies have proposed that LDR might trigger adaptive responses that promote neuroprotection, enhancing cellular resilience against radiation-induced damage [19,20,21]. These divergent findings highlight the dose-dependent dual effects of IR on neurocognitive function: high doses induce damaging effects, while low doses may either exacerbate or, in some cases, mitigate these effects through adaptive mechanisms.

This review aims to provide a comprehensive analysis of the current understanding of IR’s impact on the CNS, focusing on the dose-dependent mechanisms through which high- and low-dose radiation affect neurocognitive function. A multi-level analytical framework will be employed, beginning with molecular-level events, such as DNA damage and oxidative stress, and progressing to cellular responses, such as neuronal apoptosis and glial activation. The review will also examine neural network alterations, including disruptions in synaptic plasticity, neurogenesis, and circuit function, and ultimately assess the behavioral consequences, focusing on cognitive impairments related to memory, learning, and executive functions. Special attention will be given to the adaptive response to LDR, with an exploration of the molecular mechanisms underlying these responses and their potential role in neuroprotection. This review aims to bridge the gap between basic research and clinical applications and guide the formulation of therapeutic strategies that could safeguard cognitive function in patients undergoing radiation therapy and those exposed to LDR in occupational or environmental settings.

## 2. The Impact of High-Dose Radiation on Neurocognitive Function

The impact of HDR on the CNS has traditionally been characterized by clear and severe biological consequences, particularly in the context of standard cranial radiotherapy [21,22,23]. However, emerging evidence suggests that under specific environmental conditions or distinct radiation qualities—such as exposure to components of Galactic Cosmic Radiation (GCR) or optimized delivery modalities—the biological outcomes may be more complex. Studies indicate that within these specific contexts, radiation may exert neutral or even positive modulatory effects rather than strictly linear toxicity [24]. Furthermore, outcomes are often contingent on factors such as dose rate; for instance, ultra-high dose rate (FLASH) radiotherapy has been shown to spare neurocognitive function compared to conventional dose rates [25]. Nevertheless, in standard clinical scenarios where cumulative doses are high and delivered at conventional rates, HDR triggers a series of biological events that progressively disrupt molecular integrity, impair cellular function, disturb neural network connectivity, and ultimately lead to behavioral dysfunction.

In this paper, we will systematically explore the mechanisms and functional consequences of HDR on the CNS through a multi-level framework, progressing from molecular responses (such as DNA damage and oxidative stress) to cellular responses (including neuronal apoptosis and glial activation), then to neural network alterations (such as synaptic plasticity changes and impaired neurogenesis), and ultimately to behavioral consequences, focusing on deficits in memory, learning, and executive function.

Importantly, while ionizing radiation can inhibit neurogenesis in part through neuroinflammatory responses [26], such effects are typically transient, with recovery occurring over time following a single exposure [27]. Thus, a single high-dose exposure is unlikely to induce persistent chronic neuroinflammation.

**Figure 1:**
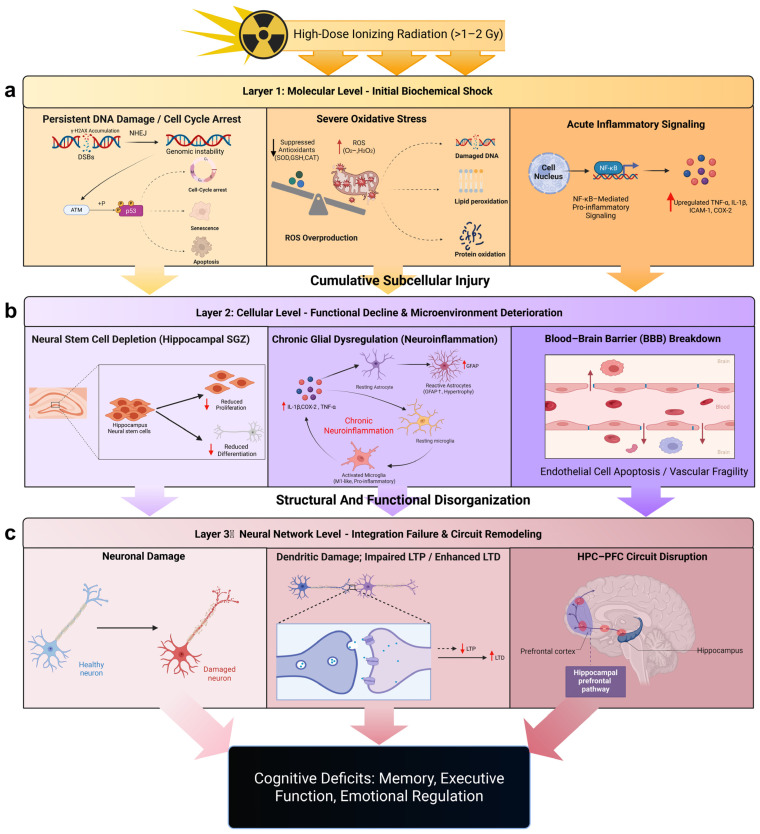
Multi-level mechanisms underlying high-dose ionizing radiation-induced neurocognitive impairment. (**a**) Layer 1, molecular-level. High-dose ionizing radiation (HDR) (>1–2 Gy) causes extensive DNA double-strand breaks (DSBs), persistent γ-H2AX accumulation, and activation of the ATM–p53 pathway, leading to cell-cycle arrest, apoptosis, and senescence. Concurrent overproduction of reactive oxygen species (ROS) results in severe oxidative stress, mitochondrial dysfunction, and depletion of antioxidant defenses (SOD, GSH, and CAT). Early inflammatory signaling is triggered through NF-κB activation, driving the upregulation of pro-inflammatory cytokines (IL-1β, TNF-α, COX-2, and ICAM-1). (**b**) Layer 2, cellular level. Long-term high-dose radiation inhibits hippocampal neurogenesis, primarily by reducing the proliferation of neural stem/progenitor cells and their capacity for neurogenic differentiation in the dentate gyrus (DG). Microglia shift toward a pro-inflammatory M1-like phenotype, while astrocytes undergo reactive gliosis (increased GFAP expression), together contributing to chronic neuroinflammation. Oligodendrocyte loss impairs myelination. Simultaneously, blood–brain barrier (BBB) integrity is disrupted due to endothelial apoptosis and vascular fragility, facilitating peripheral immune cell infiltration and amplifying local inflammatory injury. (**c**) Layer 3, network level. Cumulative molecular and cellular disturbances lead to structural and functional synaptic deficits, including reduced dendritic complexity, decreased spine density, impaired long-term potentiation (LTP), and enhanced long-term depression (LTD). Disruption of primary cognitive circuits—particularly the hippocampus–prefrontal cortex (HPC–PFC) pathway—reduces oscillatory coupling and network synchrony. Large-scale functional reorganization, including decoupling of the default mode network (DMN) from other cognitive systems, ultimately results in persistent impairments in learning, memory, executive function, and emotional regulation. Note: This figure is a conceptual schematic illustrating hypothesized pathways and mechanisms; it does not represent a quantitative model derived from specific computational data. Created in BioRender. Xu, Y. (2026), https://BioRender.com/vqazg6p.

A schematic overview of this multi-level pathophysiological cascade is illustrated in Figure 1. By elucidating the pathophysiological processes from primary biochemical reactions to complex neurobehavioral dysfunctions, we aim to provide a comprehensive understanding of radiation-induced brain injury, laying the groundwork for potential intervention strategies.

### 2.1. Initial Molecular Impact: DNA Damage, Oxidative Stress, and Activation of Inflammatory Signaling

#### 2.1.1. DNA Damage

The most direct effect of HDR on cells is DNA damage [22]. Radiation triggers the generation of free radicals during the radiolysis of water and the destruction of biomolecules, which can lead to various structural damage [23]. Among these, the most destructive are DNA double-strand breaks (DSBs). DSBs can occur directly through ionization of the DNA molecule or indirectly through radiation-induced production of reactive oxygen species (ROS). When radiation doses exceed approximately 2 Gy, the efficiency of DSB repair by high-fidelity homologous recombination (HR) significantly declines. As a result, the classical non-homologous end joining (c-NHEJ) pathway becomes the primary repair mechanism [28], leading to genomic instability.

Under HDR conditions, radiation-induced DSBs are accompanied by a rapid accumulation of γ-H2AX at the damage sites, a hallmark of the DNA damage response [29]. Animal studies have shown that γ-H2AX foci in mouse brain tissue persist for extended periods after exposure to high doses of radiation [30], indicating that DSB-induced damage is both persistent and cumulative. Additionally, HDR activates the ataxia-telangiectasia mutated (ATM) kinase, leading to rapid phosphorylation and stabilization of p53 [29], which, in turn, triggers p53-dependent cell-cycle arrest, apoptosis, or senescence [31]. Notably, neurons have relatively weak DNA repair capacities compared to other cell types. As a result, they are more prone to the accumulation of DSB signals and sustained activation of the p53 pathway following radiation exposure. This limited repair capacity may contribute to increased radiation sensitivity in the CNS [32,33,34]. The cumulative DNA damage in neurons, especially in regions such as the hippocampus (HPC), impairs neurogenesis and synaptic plasticity, which are critical for cognitive function, ultimately resulting in long-term cognitive deficits [35,36].

#### 2.1.2. Oxidative Stress

HDR disrupts the dynamic balance between ROS generation and elimination, leading to oxidative stress. This imbalance in ROS levels is a primary mechanism by which radiation causes cellular damage. ROS, byproducts of normal cellular metabolism, accumulate to deleterious levels following IR exposure [37]. These elevated ROS levels initiate a cascade of oxidative events that compromise key cellular macromolecules, including lipids, proteins, and DNA, resulting in lipid peroxidation, membrane rupture, protein aggregation, and DNA strand breaks. Specifically, ROS-mediated mitochondrial dysfunction leads to a loss of mitochondrial membrane potential, which inhibits ATP synthesis, further impairing cellular energy homeostasis and survival [37,38,39,40].

The brain, due to its high metabolic demand and limited regenerative capacity, is particularly susceptible to oxidative damage induced by IR. Among the brain regions, the HPC plays a crucial role in memory formation and learning and thus exhibits a unique vulnerability to radiation-induced oxidative stress. Research has demonstrated that after doses exceeding 2 Gy, key antioxidant enzymes such as superoxide dismutase (SOD), glutathione (GSH), and catalase (CAT) exhibit significantly reduced activity, impairing the HPC’s ability to neutralize ROS [41]. Furthermore, studies have shown that within 24 h of irradiation, the activities of Cu/Zn-SOD and Mn-SOD in the HPC and cerebral cortex decrease in a dose-dependent manner (2–5 Gy), with a more pronounced reduction observed in the HPC [42]. This limited antioxidant buffering capacity renders the HPC particularly susceptible to oxidative injury and likely contributes to radiation-induced cognitive deficits.

#### 2.1.3. Early Inflammatory Signaling

During the early stages of radiation-induced injury, transcription factors such as nuclear factor kappa-light-chain-enhancer of activated B cells (NF-κB) are significantly activated, leading to aberrant expression of pro-inflammatory cytokines [43,44,45]. This activation is often accompanied by an increase in apoptosis [40,46]. HDR exposure typically results in a substantial upregulation of several pro-inflammatory cytokines in the brain, including tumor necrosis factor-α (TNF-α), interleukin-1β (IL-1β), intercellular adhesion molecule-1 (ICAM-1), and cyclooxygenase-2 (COX-2) [44,47,48,49]. The accumulation of these factors rapidly triggers an acute inflammatory response within the CNS, exacerbating the damage caused by radiation exposure.

Microglia, the primary immune cells in the CNS, transition from a resting state to an activated M1 phenotype during this inflammatory response. In this activated state, microglia release a variety of pro-inflammatory mediators, further amplifying the inflammatory cascade [50]. Prolonged microglial activation may lead to suppression of neurogenesis, reduced synaptic plasticity, and subsequent cognitive dysfunction [35,51,52]. In addition, prolonged activation of NF-κB, COX-2, and inducible nitric oxide synthase (iNOS) further exacerbates neural injury by reinforcing inflammatory signaling and secondary oxidative damage [53,54,55]. In addition to these molecular changes, the extracellular matrix undergoes remodeling, with increased expression of matrix metalloproteinases (MMPs), which degrade the extracellular matrix and disrupt the integrity of the blood–brain barrier (BBB) [56,57]. This compromise facilitates infiltration of peripheral immune cells into the CNS, thereby sustaining chronic inflammation. If unresolved, persistent inflammatory signaling establishes a vicious cycle of neuronal injury, synaptic dysfunction, and progressive cognitive decline, particularly affecting memory and learning processes [58,59,60].

Preclinical studies have shown that late-onset radiation-induced brain damage, including cognitive dysfunction, is often driven by both acute and chronic oxidative stress and inflammatory responses. These processes interact in complex ways, with initial inflammatory damage triggering adaptive neuroprotective responses that are later overwhelmed, contributing to long-term neurocognitive impairments. This dual effect, in which inflammation initially exacerbates damage but later induces protective responses, emphasizes the critical role of dose and timing in determining the ultimate impact of radiation on the brain [61,62].

### 2.2. Consequences at the Cellular Level: Functional Cell Depletion and Deterioration of the Supportive Environment

Following the accumulation of molecular damage, cellular structural and functional alterations constitute the central mechanisms by which HDR impairs the CNS. At this stage, HDR triggers a complex cascade of cellular disturbances, including inhibition of neurogenesis, maladaptive glial activation, and progressive breakdown of the BBB. This triad collectively undermines neural homeostasis and intercellular communication within the CNS. Radiation-induced inhibition of neural stem and progenitor cell proliferation—particularly within the hippocampal dentate gyrus (DG)—reduces the brain’s regenerative potential and plasticity. Meanwhile, aberrant activation of astrocytes and microglia leads to chronic neuroinflammation, oxidative stress, and excitotoxicity, which together exacerbate neuronal vulnerability. The breakdown of the BBB further allows peripheral immune cells and inflammatory mediators to infiltrate the CNS, disrupting metabolic balance and accelerating deterioration of the microenvironment. Altogether, these cellular-level alterations not only weaken the regenerative and adaptive capacities of neural tissue but also create a self-perpetuating cycle of functional cell depletion and supportive niche degradation, thereby laying the groundwork for subsequent neural circuit disruption and cognitive dysfunction.

#### 2.2.1. Neurogenic Inhibition

In rodent models, the HPC plays a pivotal role in the acquisition, consolidation, and retrieval of information [63]. Structurally, it comprises the DG, cornu ammonis 3 (CA3), and cornu ammonis 1 (CA1) regions, among which the subgranular zone (SGZ) of the DG serves as one of the principal sites of adult neurogenesis in the mammalian brain. Within this niche, neural stem cells (NSCs) possess the capacity for self-renewal and differentiation into neurons, astrocytes, and oligodendrocytes [64,65,66]. The maintenance of hippocampal neurogenesis is critically dependent on a specialized microenvironment, in which endothelial cells and astrocytes jointly regulate proliferation, lineage commitment, and survival of newborn neurons [67,68].

Experimental evidence demonstrates that hippocampal irradiation results in a dose-dependent depletion of NSCs, accompanied by reduced proliferative activity of surviving stem cells and a marked decline in their neuronal differentiation potential [35,69,70,71]. The SGZ is particularly radiosensitive, even at clinically relevant therapeutic doses. Both single and fractionated whole-brain irradiation in adult mice and rats lead to a pronounced reduction in newly generated neurons—whether immature or mature—in the DG, which directly correlates with impaired HPC-dependent spatial learning and memory performance [35,51,72].

Notably, aged rats fail to exhibit radiation-induced reductions in neurogenesis but still show significant cognitive deficits [73,74]. Conversely, the opposite dissociation has also been observed: significant suppression of neurogenesis does not inevitably precipitate cognitive impairment [75,76]. This observation suggests that the detrimental effects of high-dose irradiation on cognitive function are not solely attributable to impaired neurogenesis but may also involve secondary mechanisms such as glial dysfunction, vascular compromise, and altered synaptic plasticity [72,77,78]. Together, these findings underscore that neurogenic inhibition constitutes a key, yet not exclusive, cellular pathway mediating the dose-dependent dual effects of IR on neural cognition.

#### 2.2.2. Glial Responses

Glial cells exhibit complex and sustained responses following exposure to HDR. Oligodendrocytes are particularly vulnerable, undergoing apoptosis or differentiation arrest after irradiation, resulting in impaired myelination and subsequent white matter injury [79,80]. However, some studies have shown that under localized high-dose radiation conditions (e.g., 10 Gy), activation of neural stem cells in the subventricular zone (SVZ) and their migration to lesion sites can promote remyelination [81]. Despite these potential compensatory mechanisms, in the context of standard cranial radiotherapy, the predominant outcome remains the compromise of axonal conduction and neural signal transmission, thereby contributing to the deterioration of cognitive processing efficiency.

Microglia and astrocytes are recognized as the principal mediators of post-irradiation neuroinflammatory reactions within the CNS [46]. Microglia play an essential role in immune surveillance, brain development, and synaptic remodeling [82,83]. In their resting state, microglia display a highly ramified morphology and continuously monitor the surrounding microenvironment. Following radiation-induced stress, they undergo marked activation, adopt a pro-inflammatory phenotype (M1 type), and release cytokines and ROS to eliminate damaged or apoptotic cells [84,85,86,87]. While this response is generally viewed as a defense mechanism, its outcomes are highly context-dependent. Notably, some studies suggest that moderate stimulation of microglial activation can exert neuroprotective effects, thereby improving cognitive function under neurodegenerative disease conditions [88]. While this transient activation can be protective, prolonged or uncontrolled activation leads to excessive neurotoxicity, secondary tissue injury, and disruption of neural connectivity [89,90].

Astrocytes, in turn, undergo pronounced reactive gliosis in response to harmful stimuli. This phenomenon is characterized by cell body hypertrophy, elongation of cellular processes, and upregulation of glial fibrillary acidic protein (GFAP) [91]. High-dose irradiation (20–45 Gy) has been shown to induce persistent astrocytic proliferation, which can persist for more than 1 year after exposure [92,93,94]. Accumulating evidence suggests a strong association between radiation-induced astrocytosis and cognitive dysfunction in patients with radiation-induced brain injury [95].

Sustained activation of microglia and astrocytes constitutes a hallmark of chronic neuroinflammation. This prolonged inflammatory state fosters a vicious cycle of neuronal damage: the continuous release of pro-inflammatory mediators aggravates neuronal stress and death, which, in turn, further amplifies glial activation and tissue degeneration. Collectively, these chronic glial responses contribute substantially to the long-term neurotoxic and neurodegenerative consequences of HDR, providing a cellular basis for persistent cognitive deficits [16,96].

#### 2.2.3. Disruption of the Blood–Brain Barrier

The BBB, composed of brain endothelial cells, the basal lamina, and astrocytic end-feet, serves as a highly selective permeability barrier that maintains the homeostasis of the CNS [97]. This structural and functional barrier tightly regulates the exchange of molecules between the bloodstream and neural tissue, thereby protecting the brain from peripheral toxins and immune components [98].

Exposure to HDR leads to profound BBB disruption. Doses exceeding 5 Gy are sufficient to induce endothelial apoptosis and microvascular injury, which, in turn, contribute to cognitive dysfunction [99]. Once BBB integrity is compromised, peripherally derived macrophages and infiltrating leukocytes can enter the brain parenchyma, expressing adhesion molecules such as C-C chemokine receptor type 2 (CCR2) and ICAM-1 that promote inflammatory cascades [100,101]. Radiation also increases vascular fragility, predisposing surviving endothelial cells to secondary DNA damage and premature senescence, further weakening the cerebrovascular network [102].

BBB permeability changes display a dynamic pattern over time. Studies have shown that a single whole-brain dose of 20–60 Gy produces a marked but transient increase in BBB permeability within days, followed by partial recovery over several weeks [103,104]. Other findings indicate that permeability peaks approximately 1–1.5 months post-irradiation, then gradually returns toward baseline [105]. During this transient yet critical period of barrier dysfunction, peripheral immune infiltration and microglial activation amplify neuroinflammatory signaling, initiating a “damage–inflammation–redamage” cycle that exacerbates tissue injury and neuronal stress [106,107].

Persistent impairment of BBB function is therefore considered a key contributor to chronic neuroinflammation and progressive cognitive decline following cranial irradiation. Its breakdown not only facilitates the entry of inflammatory cells but also disrupts the tightly regulated neural microenvironment, ultimately undermining neuronal survival and network stability.

### 2.3. Network-Level Dysregulation: From Synaptic Dysfunction to Circuit Remodeling Failure

Following the molecular and cellular insults induced by HDR, disturbances extend to higher organizational levels of the CNS. The coordinated integration of neuronal activity across circuits and brain regions—fundamental to efficient information processing and cognitive performance—is markedly impaired. Such disruption reflects not only the cumulative impact of synaptic and glial dysfunction but also the brain’s diminished capacity to maintain structural and functional connectivity. At the network level, high-dose irradiation alters both local synaptic organization and global communication dynamics. Synaptic density and transmission efficacy decline, leading to weakened signal propagation within hippocampal and cortical pathways. These regional changes subsequently impair the function of key neural circuits that support learning, memory, and executive functions. With progressive damage, compensatory mechanisms within the brain fail to re-establish coherent activity patterns, ultimately resulting in maladaptive network remodeling and global integration deficits.

#### 2.3.1. Synaptic Structural and Functional Alterations

In recent years, as memory has increasingly been understood to be encoded by changes in synaptic strength, interest has grown in investigating how IR affects neuronal structure and synaptic function, particularly in the HPC. High-dose irradiation has been shown to induce morphological alterations in hippocampal dendritic neurons, including reduced dendritic complexity and spine density [108]. These structural modifications are paralleled by functional impairments at the synaptic level. In vitro electrophysiological studies have demonstrated that irradiation suppresses long-term potentiation (LTP) while enhancing long-term depression (LTD) in the DG of the HPC [109]. LTP represents a fundamental cellular mechanism underlying learning and memory. In contrast, LTD is associated with synaptic weakening. This bidirectional alteration reflects an overall decline in synaptic plasticity and signal integration capacity [110]. The cumulative effect of these changes is thought to underlie the observed deterioration in HPC-dependent cognitive performance following cranial irradiation.

Evidence from both clinical and experimental studies further supports a link between radiation-induced cognitive impairment and disrupted excitatory neurotransmission. Abnormal accumulation of glutamate and altered N-methyl-D-aspartate receptor (NMDAR) signaling have been implicated in these synaptic alterations. Therapeutic interventions that modulate glutamatergic signaling—such as the administration of glutamate scavengers or NMDAR antagonists (e.g., memantine)—during or after radiotherapy have been shown to partially preserve dendritic integrity and improve cognitive outcomes compared with placebo groups [111,112].

Collectively, these findings indicate that high-dose irradiation compromises the structure and function of synaptic connections, resulting in reduced dendritic complexity, impaired plasticity, and excitatory imbalance. These synaptic-level disturbances provide a crucial foundation for subsequent disruptions in neural circuit organization and higher-order cognitive decline.

#### 2.3.2. Dysfunction of Key Neural Circuits

Cranial irradiation produces a range of neurofunctional consequences, including cognitive, endocrine, and sensory impairments [113,114,115]. Because the brain is organized as an interdependent network of specialized subregions, radiation-induced damage rarely affects isolated structures but instead disrupts communication within and between neural circuits. The extent of vulnerability varies among regions depending on cellular composition, metabolic demand, and neurogenic capacity [116,117,118,119]. However, the relationship between radiation and neurogenesis is complex; although high (8.5 Gy) and moderate (~0.5 Gy) doses transiently block neurogenesis, they do not reduce neurogenic capacity and may even enhance it over time [120,121]. Despite this potential for recovery, brain regions with high synaptic density and continuous remodeling activity—such as the HPC and prefrontal cortex (PFC)—are particularly susceptible to radiation-induced injury. Among these regions, the HPC–PFC circuit represents a central pathway underlying spatial learning, memory consolidation, executive control, and emotional regulation [122,123,124,125,126,127]. The HPC contributes to the encoding and contextual organization of information, while the PFC integrates this information for decision-making, attention modulation, and behavioral planning. Radiation exposure disrupts this bidirectional communication, weakening both bottom-up information transfer from the HPC and top-down regulatory control from the PFC.

Experimental studies in rodents have revealed that the granule cell layer of the hippocampal DG is highly radiosensitive, likely due to its dependence on continuous neurogenesis and circuit renewal for maintaining cognitive flexibility [128]. Functional imaging and electrophysiological data further indicate that HDR reduces neuronal synchrony between the HPC and PFC, diminishes theta- and gamma-oscillatory coupling, and ultimately compromises the temporal coordination essential for working memory and executive processes [129]. In addition to direct hippocampal and prefrontal dysfunction, radiation also impairs the integration of other interconnected networks, including thalamic relay pathways and cortico-striatal projections that support attentional control and response inhibition [130]. Such circuit-level disorganization manifests behaviorally as slower learning acquisition, reduced memory retention, and deficits in cognitive flexibility and inhibitory control—hallmarks of radiation-induced executive dysfunction [131,132,133,134].

Collectively, these findings demonstrate that HDR compromises the integrity of key neural circuits, particularly the HPC–PFC pathway, leading to a cascading failure of network communication and executive coordination. Disruption at this level bridges cellular and synaptic alterations with large-scale network disintegration, forming a critical link between structural injury and long-term cognitive decline.

#### 2.3.3. Global Network Reorganization

At the systems level, high-dose irradiation not only disrupts local neural circuits but also induces large-scale reorganization of whole-brain functional networks. Such alterations reflect a loss of global integration and network efficiency, which collectively undermine the coordination of distributed cognitive processes. Among the affected systems, the default mode network (DMN) has emerged as one of the most consistently impaired functional networks following cranial irradiation. The DMN comprises several key regions, including the precuneus, posterior cingulate cortex, medial prefrontal cortex (mPFC), medial temporal lobe, lateral parietal cortex, and HPC [134]. This network plays a central role in internally directed cognitive functions such as implicit learning, autobiographical memory, prospection, environmental monitoring, creativity, and self-referential processing [135,136,137,138,139]. As a metabolically demanding and structurally central network, the DMN is particularly vulnerable to aging, injury, and pathological insults [140]. Altered DMN connectivity has also been identified as a convergent biomarker across various neurocognitive disorders, including Alzheimer’s disease (AD), traumatic brain injury, and post-treatment cancer-related cognitive impairment [141,142,143].

Clinical neuroimaging studies further reveal the profound impact of HDR on brain network architecture. Resting-state functional magnetic resonance imaging (rs-fMRI), a noninvasive technique for assessing functional connectivity, has consistently shown significant reductions in DMN coupling following radiotherapy or chemoradiotherapy. Dumas et al. reported that although functional connectivity within the prefrontal executive network partially recovered over time after chemotherapy, DMN disruption persisted chronically [144]. Similarly, Miao et al. observed a tendency for the DMN to become functionally segregated from other brain networks in breast cancer patients following treatment [145], while Cheng et al. identified abnormal intra-network connections among multiple DMN subregions in a comparable cohort [146]. However, it is important to acknowledge that rs-fMRI relies on the blood-oxygen-level-dependent (BOLD) signal, an indirect measure of neural activity. Given that ionizing radiation can induce vascular injury and alter neurovascular coupling, a degree of interpretational uncertainty remains regarding whether these connectivity changes reflect primary neuronal dysfunction or are confounded by underlying vascular alterations [147,148].

Collectively, these findings suggest that high-dose irradiation triggers widespread reorganization and decoupling within the brain’s intrinsic connectivity networks. The persistent disruption of DMN integrity and its impaired interaction with other large-scale networks—such as the executive control and salience networks—provides a systems-level explanation for the broad and enduring cognitive deficits observed after cranial radiotherapy [149,150].

### 2.4. Cognitive and Behavioral Outcomes

The cumulative disruptions occurring at molecular, cellular, and network levels ultimately converge in the behavioral domain, where HDR manifests as measurable impairments in cognition, emotion, and social interaction. These behavioral outcomes represent the macroscopic expression of underlying neurobiological damage, reflecting the brain’s diminished capacity to integrate, process, and adapt to information across distributed circuits. Functionally, individuals exposed to HDR often exhibit deficits across multiple cognitive dimensions—particularly in learning, memory, executive function, and affective regulation. This section will examine these manifestations in greater detail, using evidence from both animal models and clinical observations, and illustrate how radiation-induced neural damage translates into distinct patterns of behavioral and cognitive decline.

#### 2.4.1. Behavioral Manifestations in Animal Models

Across various rodent models, IR has been shown to induce dose- and time-dependent cognitive impairments, reflecting the cumulative consequences of cellular and network disruptions [151,152]. Single high-dose cranial irradiation markedly impairs the intrinsic excitability and synaptic plasticity of hippocampal CA1 pyramidal neurons, resulting in measurable behavioral deficits. In the Morris water maze test, irradiated mice typically exhibit reduced time spent in the target quadrant and fewer platform crossings, indicative of impaired spatial learning and memory performance [153,154]. In juvenile mouse models designed to simulate pediatric cranial radiotherapy, a single exposure of 8 Gy not only damages neural stem and progenitor cell populations within the subventricular zone and DG but also produces delayed-onset spatial working memory deficits in adulthood [155]. This temporal lag between cellular injury and behavioral manifestation underscores the lasting and progressive nature of radiation-induced neurocognitive dysfunction. Similarly, neonatal rats exposed to high-dose X-ray irradiation show severe spatial navigation deficits even after controlling for potential visual confounds, confirming that the observed impairments arise from central rather than peripheral dysfunction [156]. Beyond HPC-dependent tasks, other studies have reported reductions in exploratory activity, attentional control, and fear extinction following high-dose exposure, further demonstrating the broad behavioral impact of irradiation across multiple cognitive domains [157,158,159].

Collectively, HDR produces persistent, often irreversible behavioral deficits in animal models, with severity determined by multiple interacting factors, including total dose, age at exposure, and the specific cognitive tasks used for assessment. These findings establish a clear experimental foundation linking structural and functional neural damage to behavioral outcomes, providing crucial translational insights into human radiogenic cognitive decline.

#### 2.4.2. Cognitive Impairment in Clinical Populations

Over the past decades, clinical investigations have increasingly recognized that delayed radiation-induced brain injury, typically emerging between six months and several years after cranial irradiation, represents a progressive and largely irreversible neuropathological process. The predominant histopathological features include demyelination, vascular degeneration, and radiation-induced white matter necrosis, which collectively compromise neural connectivity and information processing capacity. Epidemiological evidence indicates that approximately 50–90% of cancer survivors experience measurable neurocognitive sequelae following radiotherapy, with deficits that often intensify over time and significantly impair daily functioning and quality of life [160].

This detrimental impact spans all age groups but is most pronounced in the developing brain. Even moderate doses of IR (0.1–2 Gy) administered during critical windows of neurodevelopment have been associated with persistent neurocognitive impairment. Prenatal exposure—particularly during early to mid-gestation—has been linked to intellectual delay, learning disabilities, and neurobehavioral abnormalities in offspring [160,161,162,163,164,165]. Notably, the neurodevelopmental impact may be age-dependent and influenced by the brain’s inherent plasticity. For instance, analysis of nuclear incidents revealed that prenatal exposure to an absorbed dose of ~0.4 Gy led to cognitive declines in children aged 6–7 years, yet these impairments were no longer observed by ages 10–12, suggesting the presence of significant compensatory mechanisms during maturation [166]. In pediatric cancer survivors, particularly those treated for brain tumors or acute lymphoblastic leukemia (ALL), cognitive deficits frequently emerge years after therapy and progress insidiously [167,168]. The introduction of prophylactic whole-brain irradiation, once intended to prevent CNS relapse, paradoxically led to marked declines in intelligence quotient (IQ), with younger age at exposure predicting greater long-term impairment [169,170,171,172,173]. Recent longitudinal studies further demonstrate that survivors exposed to cranial irradiation during early childhood exhibit accelerated cognitive aging and an elevated lifetime risk of dementia, suggesting that radiation not only induces developmental disruption but also predisposes individuals to premature neurodegeneration [174].

In adult populations, neuropsychological evaluations consistently reveal persistent cognitive dysfunction following high-dose cranial irradiation, particularly in domains of attention, episodic memory, and executive control [175,176,177]. However, evidence remains inconclusive regarding apparent age-dependent differences in vulnerability among mature adults. Notably, patients with skull-base tumors who receive precisely targeted radiotherapy often maintain long-term cognitive stability in the absence of tumor recurrence or radiation necrosis [178]. This observation suggests that cognitive outcomes are modulated not only by total dose but also by individual susceptibility, treatment precision, and the anatomical specificity of the irradiated regions.

Taken together, clinical evidence unequivocally demonstrates that high-dose cranial irradiation exerts sustained and cumulative effects on cognitive function. The severity and persistence of these impairments are governed by a complex interplay of factors, including age at exposure, radiation dose and distribution, neuroanatomical vulnerability, and individual differences in neural plasticity. These findings collectively reinforce the need for dose optimization, individualized treatment planning, and the development of neuroprotective interventions aimed at preserving cognitive integrity in long-term cancer survivors.

#### 2.4.3. Potential Interventions and Neuroprotective Strategies

To mitigate or prevent radiation-induced damage to the CNS, multi-level intervention strategies have been explored, primarily targeting molecular and cellular mechanisms underlying neurotoxicity. These interventions aim to attenuate oxidative stress, suppress neuroinflammation, and restore neurogenesis—processes that collectively help preserve cognitive function after high-dose irradiation.

At the molecular level, antioxidant and anti-inflammatory agents have demonstrated considerable neuroprotective potential in animal models. For instance, hydrogen-rich water has been shown to alleviate radiation-induced cognitive deficits by activating GSH metabolism and enhancing endogenous antioxidant defense pathways [179]. Likewise, nonsteroidal and steroidal anti-inflammatory drugs, cyclooxygenase (COX) inhibitors, and peroxisome proliferator-activated receptor (PPAR) agonists have been reported to attenuate radiation-induced neuroinflammation, partially restoring hippocampal neurogenesis and improving behavioral performance [26,180,181]. These findings collectively suggest that pharmacological modulation of oxidative and inflammatory cascades may help preserve neuronal integrity and delay cognitive decline.

At the cellular level, cranial irradiation profoundly suppresses hippocampal neurogenesis and damages neural stem cell (NSC) populations, changes that are closely associated with cognitive deterioration. Transplantation of NSCs has therefore emerged as a promising therapeutic avenue. Preclinical studies indicate that NSC transplantation can enhance tissue repair and promote functional recovery in various models of neurodegeneration [182,183]. However, the inflammatory microenvironment of the irradiated brain may compromise the neurogenic niche, impeding the differentiation of grafted cells into mature neurons [26,35]. Consequently, while stem cell-based interventions hold theoretical promise, their clinical translation requires rigorous investigation into host–graft interactions, immune compatibility, and long-term safety.

In clinical practice, a paradigm shift has occurred toward the adoption of hippocampal-sparing (HS) whole-brain radiotherapy techniques to mitigate or prevent radiation-induced CNS injury. Recognizing the hippocampus as a critical and radiosensitive neurogenic niche, this approach aims to minimize the radiation dose delivered to this specific region. Pivotal clinical trials have demonstrated that HS techniques significantly reduce the risk of neurocognitive failure compared to conventional whole-brain radiotherapy (WBRT) [184]. These clinical benefits are corroborated by preclinical murine models, where conformal hippocampal avoidance has been shown to mitigate behavioral deficits and preserve neuronal structural integrity compared to uniform whole-brain exposure [117]

In summary, current preclinical evidence suggests that no single therapeutic approach can fully reverse radiation-induced brain injury. Nevertheless, both pharmacological and cell-based interventions represent viable strategies for mitigating cognitive decline and promoting neural repair. Future research should emphasize mechanistic elucidation, dose optimization, combinatorial therapy design, and translational validation to facilitate the safe and effective clinical application of neuroprotective strategies against radiation-induced cognitive dysfunction.

## 3. Effects of Low-Dose Radiation on Neurocognitive Function

In contrast to the relatively well-characterized consequences of high-dose irradiation, the neurocognitive effects of low-dose IR remain poorly defined and sometimes contradictory [14]. This uncertainty arises mainly from the inconsistent operational definitions of “low dose” across studies, including variability in both total dose and dose-rate thresholds. In this review, IR doses below 100 mGy are regarded as LDR, consistent with the UNSCEAR classification. However, it should be noted that in mechanistic animal studies, low-dose exposure is often defined in a functional rather than a strictly dosimetric manner, based on the absence of overt cytotoxicity and the induction of adaptive or hormetic biological responses. Consequently, doses up to 0.3–0.5 Gy have frequently been employed in rodent models to elicit subthreshold stress responses without causing irreversible neural damage. These doses are often considered biologically equivalent to lower-dose exposures in humans due to interspecies differences in radiosensitivity, DNA repair capacity, and neurobiological resilience.

Furthermore, in these mechanistic radiobiology studies, particularly those employing small mammalian models, radiation doses higher than the formal regulatory definition are often used for pragmatic experimental reasons. To elicit measurable adaptive or excitatory biological responses—such as changes in oxidative stress markers, inflammatory signaling, or synaptic and cognitive-related endpoints—within a feasible observational time window, investigators frequently apply doses in the range of approximately 0.1–0.5 Gy. Importantly, these dose levels are selected to induce detectable adaptive responses rather than cytotoxic effects, and their use primarily reflects considerations of experimental feasibility and biological signal detectability [20].

Such dose selection should therefore be understood as a functional experimental design aimed at uncovering potential pathways and mechanisms underlying radiation adaptive responses, rather than as an attempt to establish strict dosimetric equivalence with human exposure scenarios or radiological protection dose limits. Consequently, extrapolation of findings from these animal studies to human health risk assessment or radiation protection standards must be approached with considerable caution and requires the application of complex dose- and dose-rate correction factors, as well as robust interspecies extrapolation models.

From a theoretical perspective, radiation protection frameworks since the 1970s have adopted mainly the Linear No-Threshold (LNT) model [185] to estimate potential risks associated with low-dose exposure by extrapolating from high-dose experimental data [186,187]. According to this model, radiation-induced risk increases proportionally with dose, with no safe threshold even at minimal exposure levels [188]. However, recent advances in radiobiology suggest that this oversimplified assumption fails to account for the organism’s complex homeostatic and adaptive responses to low-dose stress. Biological systems possess intrinsic DNA repair, antioxidant, and stress-response pathways that may counteract or even overcompensate for subthreshold damage. Thus, the applicability of the LNT model at low-dose levels has become increasingly controversial [189,190].

At the same time, newer epidemiological studies continue to highlight the detrimental effects of ionizing radiation, indicating increased risks of neurodegenerative conditions such as Parkinson’s disease and cerebrovascular diseases even at relatively low exposure levels [191,192]. Furthermore, evidence regarding the long-term cognitive sequelae of environmental radiation exposure remains concerning. A recent large-scale study of individuals chronically exposed to ionizing radiation following the Chernobyl nuclear disaster reported statistically significant cognitive deficits decades after exposure [193].

An alternative theoretical framework—the threshold model—posits that organisms exhibit a biologically tolerable range of radiation exposure, below which no measurable harm occurs. While specific experimental data support the presence of a damage threshold, this model also falls short in explaining the nonlinear, time-dependent biological dynamics that often characterize low-dose responses [189].

**Figure 2:**
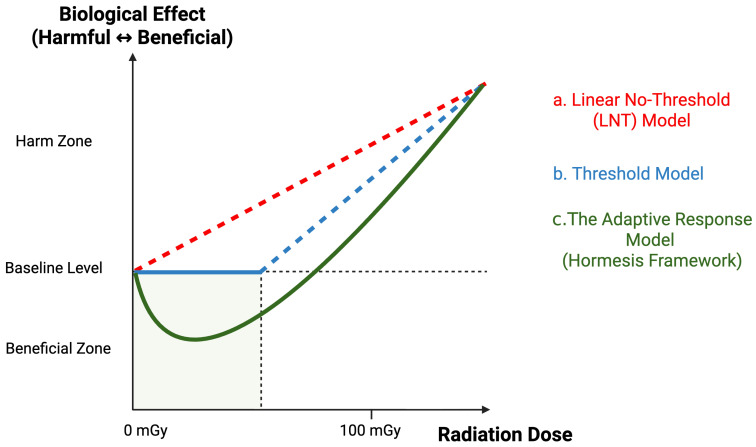
Conceptual models describing dose–response relationships of low-dose ionizing radiation. The figure illustrates three major theoretical frameworks proposed to explain the biological responses to LDR (<100 mGy). (**a**) Linear no-threshold (LNT) model: Assumes a strictly linear relationship between radiation dose and biological harm, with no safe threshold. Even minimal exposure is predicted to increase risk proportionally, reflecting a monotonic dose–response curve. (**b**) Threshold model: Proposes the existence of a biologically tolerable exposure range below a defined threshold dose, within which no detectable adverse effects occur. Beyond this threshold, radiation causes measurable, progressively increasing harm. (**c**) Adaptive response model: Suggests that LDR can activate protective mechanisms—including enhanced DNA repair, antioxidant defense, immune modulation, and mitochondrial resilience—leading to beneficial or hormetic effects. These adaptive responses may reduce subsequent damage from higher radiation challenges, generating a biphasic (beneficial-to-harmful) dose–response curve. Dose values on the x-axis are shown for schematic illustration. The commonly used 100 mGy level is included as a reference for the upper range of low-dose radiation, rather than as a definitive biological threshold. Note: This figure is a conceptual schematic illustrating hypothesized pathways and mechanisms; it does not represent a quantitative model derived from specific computational data. Created in BioRender. Xu, Y. (2026), https://BioRender.com/vqazg6p.

In the past two decades, a third paradigm—the adaptive response model—has emerged to describe phenomena in which prior exposure to LDR enhances resistance to subsequent high-dose insults. This form of radiation preconditioning has been documented across multiple species and cell types, reflecting a coordinated upregulation of antioxidant enzymes, DNA repair factors, and mitochondrial resilience mechanisms [20,194,195]. Such findings highlight the need to view radiation effects not as a monotonic continuum of harm but as a dose-dependent biphasic process integrating both injury and adaptation. These contrasting dose–response relationships of the LNT, threshold, and adaptive/hormesis models are conceptually summarized in Figure 2.

The concept of radiation adaptive response (RAR) has a long-standing history in radiobiology. The UNSCEAR has evaluated the potential adaptive effects induced by low-dose exposure and the challenges they pose to the LNT model across several reports [196,197]. At the theoretical level, researchers have proposed various frameworks, including hormetic models and biphasic dose–response relationships, to quantitatively describe this nonlinear biological phenomenon [198,199]. However, RAR remains a subject of ongoing debate within epidemiological risk estimation: while some population data suggest adaptive changes in certain health indicators following low-dose exposure, there is currently no consensus on translating these findings directly into risk adjustment factors for radiological protection [200,201].

The “priming-challenge” dose paradigm serves as a classic experimental model for studying RAR. For instance, Raper–Yonezawa-type studies have demonstrated that a priming dose of 0.01–0.1 Gy can significantly reduce the rate of chromosomal aberrations caused by subsequent high-dose radiation, with its protective window and dose dependency being well quantified [202,203]. Furthermore, chronic low-dose-rate exposure in high background radiation areas (HBRAs) provides ecological evidence for RAR; residents in these regions often exhibit enhanced DNA repair capacity and resistance to oxidative stress [204,205,206].

Importantly, threshold-like and biphasic dose–response behaviors have been formally reproduced in existing biophysical and biomathematical models that incorporate inducible DNA repair, redox regulation, and adaptive stress-response pathways. From a quantitative modeling perspective, low-dose irradiation can dynamically modify key system state variables—such as repair efficiency, oxidative damage clearance, and cellular radiosensitivity—thereby giving rise to nonlinear dose–response relationships that deviate from simple linear extrapolation [207,208,209].

Notably, RAR does not occur in isolation but is closely coupled with other stress pathways within the organism. Research indicates that radiation-induced adaptive responses can be modulated by coexisting oxidative stress or inflammatory signaling, which may either enhance or diminish the protective effect [210,211]. In a neurobiological context, this interaction is particularly critical: the activation state of microglia, mitochondrial metabolic stress, and peripheral immune signals can all influence the outcome of radiation-induced neuroprotection versus neurotoxicity [96,212,213]. Therefore, the context of multiple coexisting stressors must be considered when interpreting low-dose radiation effects in realistic and complex environments.

Correspondingly, growing experimental and epidemiological evidence has revealed that LDR does not invariably induce tissue damage and may instead trigger hormetic or beneficial biological effects under specific contexts [214,215,216]. These effects include reduced carcinogenic risk, enhanced immune surveillance, upregulation of neurotrophic factors, and improved mitochondrial redox balance. Collectively, these outcomes constitute the so-called radiation hormesis or adaptive neuroprotective response [201,217,218,219], suggesting that LDR may act as a mild stressor capable of activating endogenous defense networks, thereby strengthening resilience against subsequent neurotoxic challenges.

It should be noted that the majority of experimental evidence supporting low-dose adaptive or hormetic responses in the CNS is derived from studies using low-LET radiation, predominantly gamma rays and X-rays. In contrast, data from high-LET exposures remain limited and largely indicate deleterious outcomes even at comparably low doses.

**Figure 3:**
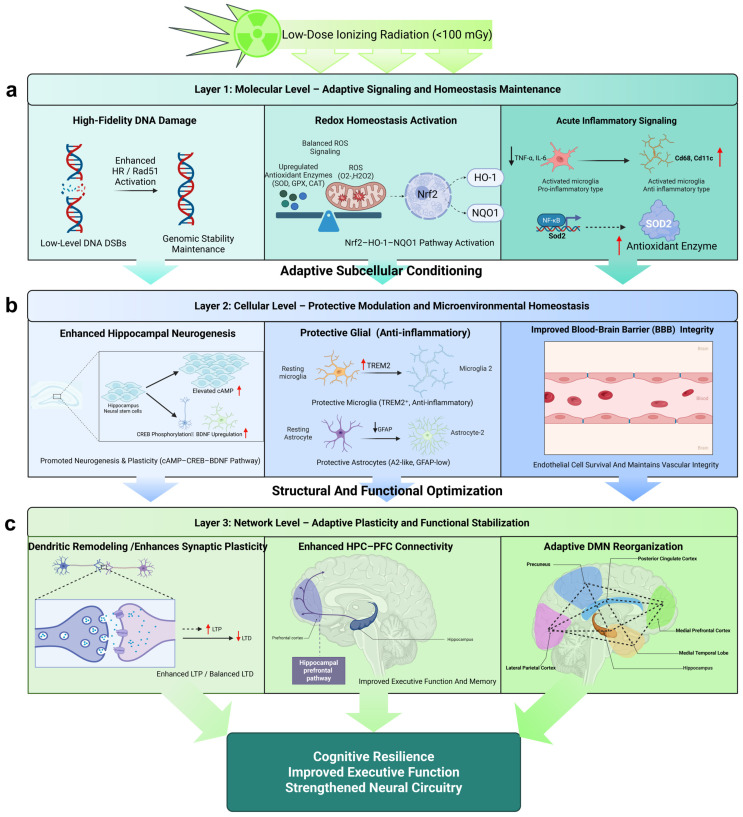
Multi-level adaptive responses to low-dose ionizing radiation. (**a**) Layer 1, molecular-level regulation. LDR activates a coordinated set of adaptive signaling pathways that enhance genomic integrity and redox homeostasis. Mild DSBs preferentially trigger high-fidelity homologous recombination (HR) repair, as indicated by Rad51 upregulation, promoting long-term genomic stability. Transient increases in ROS induce antioxidant responses via activation of the Nrf2–HO-1–NQO1 pathway, as evidenced by elevated SOD, GPX, and CAT activity. Low-dose exposure also modulates inflammatory signaling: NF-κB activation drives controlled expression of immune-related genes (e.g., *Sod2*), while microglia exhibit both transient pro-inflammatory (Cd68^+^, Cd11c^+^) and compensatory anti-inflammatory phenotypes. Together, these processes reinforce cellular homeostasis and protect against subsequent oxidative or genotoxic stress. (**b**) Layer 2, cellular-level adaptations. LDR promotes neurogenesis and enhances neuronal plasticity by activating intracellular signaling cascades, including cAMP elevation and CREB phosphorylation, thereby increasing brain-derived neurotrophic factor (BDNF) expression. Neural stem/progenitor cells within the HPC exhibit improved survival and differentiation under subthreshold stress. Glial cells undergo protective phenotypic shifts—microglia polarize toward an anti-inflammatory, reparative TREM2^+^ phenotype, while astrocytes adopt GFAP-low, neurotrophic A2-like features. Concurrently, low-dose exposure supports structural and functional repair of the BBB, stabilizing neurovascular homeostasis. These cellular adaptations collectively strengthen endogenous defense networks and facilitate neural resilience. (**c**) Layer 3, neural network-level plasticity. At the systems level, low-dose irradiation enhances synaptic plasticity and stabilizes large-scale neural network function. Electrophysiological data indicate improved LTP and balanced LTD, supporting circuit-level stability. Strengthening of the HPC–PFC pathway enhances executive function and memory integration. Functional network analyses reveal adaptive reorganization within the DMN, including increased connectivity across the precuneus, posterior cingulate cortex, medial temporal lobe, and mPFC. These network-level changes contribute to improved cognitive robustness and compensatory functional capacity following low-dose exposure. Note: This figure is a conceptual schematic illustrating hypothesized pathways and mechanisms; it does not represent a quantitative model derived from specific computational data. Created in BioRender. Xu, Y. (2026), https://BioRender.com/vqazg6p.

Building on these theoretical and empirical insights, we aim to systematically explore the multi-level adaptive responses elicited by LDR—from molecular signaling pathways and cellular physiology to neural circuit remodeling and cognitive–behavioral outcomes, as conceptually summarized in Figure 3. The goal is to establish an integrated conceptual framework describing the dose-dependent dual effects of IR on the brain—linking early molecular perturbations to long-term neurocognitive adaptation and resilience.

### 3.1. Molecular-Level Regulation

#### 3.1.1. Dose-Dependent Regulation of DNA Damage and Repair Systems

At the molecular level, LDR exerts complex, bidirectional regulatory effects, with its biological consequences determined by an interplay of factors including dose, exposure duration, radiation quality, and cellular context. Within a specific range, such exposure can act not as a damaging stimulus but as a mild stressor that activates endogenous protective systems, thereby maintaining a balance between damage and defense. LDR may induce transient oxidative stress and limited DNA perturbations that do not exceed cellular repair capacity, thereby triggering compensatory signaling pathways rather than cytotoxic cascades. These include enhanced DNA repair mechanisms, redox regulation, mitochondrial homeostasis, and modulation of inflammatory signaling, all of which contribute to maintaining neural integrity and eliciting an adaptive protective effect.

Studies using γ-H2AX and Rad51 as biomarkers for DSBs and HR, respectively, have revealed that the number of DSBs increases linearly with radiation dose. In contrast, subsequent activation of DNA repair systems shows a dose-dependent pattern of regulation. At lower doses, cells preferentially activate the HR pathway over the error-prone non-homologous end joining (NHEJ) [189], reflecting a shift toward high-fidelity repair. Because HR requires more time to complete but preserves genomic integrity, this low-dose-induced repair bias contributes to long-term genomic stability. It initiates an adaptive molecular response that enhances resilience to later oxidative or genotoxic stress. This concept is supported by evidence demonstrating that cells pre-exposed to LDR exhibit fewer DSBs when challenged with higher doses [220,221,222,223,224,225].

Yin et al. reported that whole-body exposure to 0.1 Gy in mice triggered significant transcriptional changes in the brain associated with DNA repair and cell survival pathways, suggesting a neuroprotective potential [226]. Similarly, in a murine model intermittently exposed to CT-level radiation over 10 weeks, the number of DSBs following a high-dose challenge was significantly lower than in controls [227]. Beyond these acute observations, experimental evidence suggests that mice under more prolonged low-dose irradiation exhibit extended lifespans, indicating that the systemic benefits of radiation hormesis may translate into improved long-term survival [228]. Chronic low-dose irradiation has also been shown to reduce baseline DSB levels below those of unexposed animals, indicating that low-dose exposure can “precondition” or “pre-activate” DNA repair capacity, which may represent a core molecular mechanism underlying the radiation adaptive response [229].

Although traditional research focuses on genomic integrity, some studies indicate that at lower doses, the primary biological targets may shift toward non-genomic structures, including damage to small membrane structures or alterations in functional connectivity. These perspectives suggest that even in the absence of significant DNA damage or overt cell death, radiation-induced cognitive decline may stem from disruptions in synaptic efficacy and network topology. However, it should be noted that much of the evidence supporting these non-genomic targets is derived from studies employing high-LET radiation, the effects of which may differ to some extent from those of low-LET exposure [230,231].

Taken together, these findings indicate that DNA repair systems exhibit a distinct dose-dependent bifurcation: LDR favors error-free repair and adaptive genome stabilization, whereas high-dose exposure overwhelms repair capacity, leading to persistent DNA damage, chromosomal aberrations, and apoptosis. This mechanistic divergence forms a key molecular basis for the dual dose-dependent effects of IR, balancing genotoxic injury and adaptive neuroprotection.

#### 3.1.2. Activation of Redox Balance and Antioxidant Defense Systems

ROS play a dual role in cellular regulation. At elevated levels, ROS are known to induce DNA damage, cell-cycle arrest, and apoptosis [232]. However, at low doses, radiation-induced ROS can trigger beneficial cellular responses, promoting adaptation and even conferring radiation resistance in both irradiated and bystander cells [211,233]. Studies have demonstrated that LDR leads to a modest increase in ROS, which activates the intracellular antioxidant defense systems, triggering an adaptive response. This response includes the upregulation of key antioxidant enzymes, such as SOD, CAT, and glutathione peroxidase (GPX), reflecting a shift in cellular redox balance towards homeostasis [234].

At the molecular level, this mild oxidative stress is not merely a byproduct of radiation exposure but a crucial signal that initiates adaptive mechanisms to restore cellular function. A key pathway involved is the activation of nuclear factor erythroid two-related factor 2 (Nrf2), a transcription factor central to regulating the antioxidant response. Upon activation, Nrf2 translocates to the nucleus, where it induces the expression of critical antioxidant genes, including heme oxygenase-1 (*HO-1*) and *NAD(P)H*: quinone oxidoreductase 1 (*NQO1*) [235,236,237]. These enzymes facilitate ROS detoxification and promote cellular repair, aiding in the restoration of redox balance and protecting cells from further oxidative damage.

Moreover, Nrf2 is not the only redox-sensitive pathway that contributes to the cellular response to LDR. Other signaling molecules, such as p53 and NF-κB, play a critical role in regulating cellular responses to oxidative stress. These pathways modulate apoptosis, inflammation, and cell survival, providing a comprehensive network for cellular adaptation. Specifically, NF-κB activation facilitates anti-inflammatory responses that help mitigate chronic inflammation often observed following HDR exposure [238,239,240].

Collectively, these mechanisms highlight the role of ROS not only as harmful agents but also as signaling molecules that regulate cellular functions. The activation of antioxidant defense systems through pathways like Nrf2, p53, and NF-κB forms a coordinated network that restores redox balance and reduces oxidative damage. This adaptive regulation is essential for maintaining cellular homeostasis, protecting against radiation-induced damage, and enhancing long-term cellular resilience.

#### 3.1.3. Inflammatory Response and Neuroimmune Regulation

In contrast to the neuroinflammation typically induced by HDR, LDR can, under specific conditions, exert anti-inflammatory effects. LDR has been shown to inhibit the release of pro-inflammatory cytokines and to polarize microglia toward an anti-inflammatory phenotype (M2 type). For example, following 100 mGy whole-body irradiation, the number of activated microglia in the mouse brain increases, accompanied by upregulation of pro-inflammatory markers such as Cd68 and Cd11c, suggesting immune system activation [241]. LDR has also been shown to induce microglial activation, which mediates immune responses [242]. Additionally, LDR can activate the NF-κB signaling pathway, which, through feedback regulation, induces the transcriptional activation of antioxidant enzymes such as SOD2, thereby enhancing cellular antioxidant capacity [225].

Experimental studies indicate that early exposure to 0.1 Gy LDR can significantly reduce inflammation in the HPC and cortex induced by subsequent 2 Gy HDR [241]. In an experimental autoimmune encephalomyelitis (EAE) mouse model of multiple sclerosis, repeated γ-irradiation at 0.5 Gy has been shown to alleviate neuroinflammation by suppressing pro-inflammatory cytokines such as TNF-α and IL-6 and promoting the recruitment of regulatory T cells [243,244]. Similar effects have been observed in genetic autoimmune disease mouse models, where 5 weeks of continuous exposure to 0.35 mGy/h and 1.2 mGy/h gamma irradiation resulted in significantly extended lifespan and reduced brain inflammation [245].

Collectively, LDR can induce significant adaptive regulatory effects at the molecular level by promoting error-free DNA repair, activating antioxidant defense systems, and suppressing excessive inflammatory responses. Although these mechanisms have been validated in various cellular models, further systematic investigation is needed to better understand the specific regulatory mechanisms of the innate immune system in the brain and the associated dose–effect relationships.

### 3.2. Subtle Cellular-Level Balance

At the cellular level, LDR also exhibits dose-dependent bidirectional regulatory effects. Unlike HDR, which predominantly induces cellular damage and suppresses neurogenesis, LDR may, under certain conditions, activate intrinsic cytoprotective pathways, thereby eliciting opposing biological outcomes.

#### 3.2.1. Dose-Dependent Response of Neural Stem Cells and Neurogenesis

In contrast to the well-documented inhibitory effects of high-dose irradiation on hippocampal neurogenesis, LDR can, under specific conditions, elicit a stimulatory response. At extremely low exposure levels, it may exhibit an “excitatory effect”, slightly enhancing the proliferation and differentiation of NSCs, whereas at somewhat higher doses—though still within the low-dose range—the effect tends to shift toward suppression. Studies have demonstrated that LDR can enhance hippocampal neurogenesis, improve learning and memory performance, and support cognitive health [246,247]. In mouse models, LDR (0.075 Gy) exposure increased cAMP levels and the cAMP/cGMP ratio in the hypothalamus, a change associated with neuronal sprouting, axonal elongation, and synaptic remodeling [248,249]. These findings imply that LDR may activate intracellular signaling cascades. Yahyapour et al. [250] reported in a Parkinson’s disease (PD) mouse model that LDR significantly reduced oxidative stress and neuronal apoptosis, indicating potential neuroprotective effects. Similarly, another study showed that 0.3 Gy irradiation promoted NSC proliferation, enhanced hippocampal neurogenesis, and improved spatial learning, likely via activation of the Wnt/β-catenin signaling pathway [251]. Activation of this pathway may also intersect with other regulatory networks, such as Notch and PI3K/Akt, further supporting neurogenic and survival-promoting outcomes under sub-toxic stress conditions.

However, not all studies have reported beneficial effects. Some findings indicate that LDR may still induce subtle cellular dysfunction, depending on developmental timing and cumulative exposure. For instance, 0.05 Gy irradiation significantly increased the expression of synaptic markers such as Synapsin 1, Synaptophysin, SAP97, Debrin 1, and PSD95 [252], which may reflect aberrant synaptic remodeling rather than functional enhancement. This could lead to altered synaptic plasticity and learning impairments. Likewise, 0.5 Gy exposure has been associated with neural precursor cell dysfunction and cognitive deficits [253].

#### 3.2.2. Phenotypic Transformation of Glial Cells and Neuroimmune Regulation

LDR affects not only neurons but also modulates the activation dynamics of glial cells, which play essential roles in maintaining neural homeostasis and mediating immune responses in the brain. In contrast to HDR, which has been reported to induce pro-inflammatory M1-type microglia and neurotoxic A1-type astrocytes (reactive phenotype), low-dose exposure tends to bias toward a shift toward protective phenotypes—microglia polarizing to the anti-inflammatory M2 state and astrocytes differentiating toward the neurotrophic A2-type astrocytes (protective phenotype) [254,255]. Such phenotype switching has been proposed as a potential adaptive response through which glial cells may participate in the restoration of tissue homeostasis following mild stress.

M2-type microglia exert transient neuroprotective and anti-inflammatory effects, facilitating remyelination, phagocytic clearance, and tissue repair, while attenuating secondary inflammatory cascades [256,257,258]. In an AD mouse model, LDR upregulated Trem2 expression, which, in turn, promoted M2 polarization, suppressed Aβ-induced neuroinflammation, and alleviated memory deficits [259]. These observations suggest that moderate redox perturbation and cytokine feedback at low radiation doses may favor anti-inflammatory reprogramming, allowing microglia to support neuronal recovery rather than perpetuate damage. Morphological studies further confirm this pattern: 0.063 Gy irradiation induced a highly ramified, surveillance-oriented microglial morphology, typical of physiological remodeling, whereas 0.5 Gy exposure caused progressive de-ramification and transformation into an amoeboid, phagocytic state, characteristic of chronic activation [260].

Astrocytes exhibit a similarly dose-dependent response to LDR. Studies show that following exposure to 0.125–0.5 Gy, astrocytes in the DG and CA1 regions displayed reduced branching complexity, process length, and density within 24 months [260]. In contrast, lower doses did not elicit comparable structural alterations. As the radiation dose increases, neurotoxic A1 astrocytes—characterized by complement component 3 (C3) expression [261]—become more prevalent, while neuroprotective A2 astrocytes are preferentially induced under specific low-dose conditions [260]. This balance between A1 and A2 astrocyte phenotypes appears to be mediated by cytokine signaling, notably through the IL-10 and Transforming Growth Factor-beta (TGF-β) pathways, which are upregulated under subthreshold oxidative stress [262,263].

It is noteworthy that fractionated low-dose irradiation studies have shown that even low-dose exposures can elicit persistent neuroinflammatory responses during the early post-irradiation period, manifested as increased microglial activation and reactive astrogliosis, which subsequently have been associated with impaired hippocampal neurogenesis [91]. In certain clinical contexts, repeated low-dose exposures have been reported to accumulate biological effects that, under prolonged or high-frequency exposure conditions, may shift from adaptive responses toward potentially adverse outcomes [264]. These findings suggest that the glial response to LDR is inherently bidirectional, governed by a complex interplay of dose magnitude, temporal pattern, and microenvironment.

#### 3.2.3. Functional Alterations of the Blood–Brain Barrier and Potential Mechanisms

HDR-induced disruption of the BBB has long been recognized as a central pathological mechanism underlying acute radiation syndrome and delayed radiation-induced brain injury. However, an expanding body of evidence now indicates that even moderate and low-dose cranial irradiation can alter BBB permeability in a dose-dependent and age-dependent manner. These findings broaden the traditional view of radiation-induced neurovascular injury and suggest that exposures well below cytotoxic levels can subtly modify the physiological barrier function of cerebral microvessels.

Experimental data from adult mouse models demonstrate that cranial irradiation across a wide dose range can increase cerebrovascular permeability. While high-dose exposure typically leads to more pronounced and long-lasting BBB disruption, changes induced by moderate or low doses are often partially reversible within several months. These observations suggest an intrinsic capacity of the cerebral vasculature to engage spontaneous repair and adaptive compensation under sublethal stress conditions [265].

Recent studies further reveal that LDR may, under certain circumstances, trigger compensatory or adaptive responses that transiently confer protective effects on the BBB. Mechanistically, LDR has been shown to generate small amounts of ROS that act as signaling intermediates to activate the Nrf2/HO-1 pathway, thereby inducing downstream antioxidant genes, including *HO-1*, *NQO1*, and *SOD* [266,267]. These enzymes help neutralize excessive ROS and protect endothelial cells from oxidative injury. In parallel, LDR has been reported to activate the Wnt/β-catenin pathway, promoting the transcription and assembly of tight junction proteins, including Claudin-5, Occludin, and ZO-1, ultimately strengthening the structural integrity of the BBB and limiting paracellular leakage [251].

At the immunological level, low-dose irradiation tends to suppress the pro-inflammatory polarization of microglia and astrocytes, thereby reducing the release of BBB-disrupting cytokines such as TNF-α and IL-1β [59,268]. Additionally, LDR may enhance the secretion of neurotrophic factors, such as brain-derived neurotrophic factor (BDNF), which could contribute to a microenvironment supportive of BBB repair. Together, these multi-layered mechanisms are proposed to place the BBB in a state of relatively enhanced defensive readiness, which may increase its resilience against subsequent insults such as HDR, ischemia, or oxidative stress.

In summary, LDR does not simply act as a detrimental factor; instead, it can engage multiple compensatory signaling pathways that may induce adaptive protective responses within the BBB. These insights provide a potential theoretical foundation for exploring low-dose irradiation as a “preconditioning” strategy to mitigate neurological damage, which requires further experimental and translational validation.

### 3.3. Neural Networks and Cognitive–Behavioral Implications

The effects of LDR on the nervous system generally do not manifest as acute tissue damage or overt behavioral abnormalities. Instead, they may involve subtle modulations of neural circuits and network remodeling, which gradually accumulate into subclinical functional changes. These alterations may reflect a transient adaptive enhancement or potentially indicate early signs of an imbalance in neural function.

#### 3.3.1. Bidirectional Regulation of Synaptic Plasticity Under Low-Dose Radiation

LTP and LTD interact at the neural network level, regulating synaptic strength and thereby maintaining circuit stability and learning capacity. This process, referred to as homeostatic synaptic plasticity (HSP), acts as a critical negative feedback mechanism that allows neurons to protect themselves from excessive excitation or inhibition [269]. As a fundamental process underlying neural circuit function, synaptic plasticity is bidirectionally regulated by LDR, with research indicating that LTP may be enhanced, suppressed, or show no significant change under these conditions.

Studies have demonstrated that chronic low-dose neutron irradiation can reduce neuronal excitability and impair LTP in both the HPC and the mPFC [270]. In juvenile rats, significant reductions in both LTD and LTP were observed following irradiation [271]. In contrast, other research has reported that LDR can increase excitability and synaptic efficacy in wild-type mice [272], and 28Si ion irradiation enhances synaptic plasticity in the CA1 region of the HPC [273].

The observed discrepancies in these findings are likely due to several key parameters, including total absorbed dose, dose rate, and time points post-exposure. This high degree of variability, based on current evidence, highlights the inherent complexity of studying the neural effects of LDR. It underscores the need to carefully interpret findings within specific experimental conditions. The interaction between these factors suggests that LDR may elicit a range of adaptive and maladaptive responses, dependent on the precise nature of exposure.

#### 3.3.2. Vulnerability of the Hippocampal–Prefrontal Cortex Circuit and Cognitive Regulation

The HPC–PFC circuit, a principal neural network supporting learning, memory, and executive control, exhibits high sensitivity to radiation exposure across the entire dose spectrum [108,269]. While the detrimental effects of HDR on this circuit are well established, emerging evidence indicates that even low-dose exposure can induce subtle yet functionally significant alterations in synaptic transmission and network integration. Experimental findings have shown that both mice and rats subjected to chronic mixed-field neutron exposure at a dose rate of 1 mGy/day for six months exhibited disrupted HSP within the hippocampal–cortical pathway, leading to deficits in associative memory formation and impaired long-range connectivity between the HPC and PFC [269]. Such chronic low-dose exposure may alter oscillatory synchrony and phase coupling—mechanisms thought to be important for efficient information transfer across distributed cortical networks. In AD mouse models, the cognitive effects of low-dose radiotherapy (LD-RT) have attracted considerable interest. Although findings remain heterogeneous—with some studies reporting reduction in amyloid-β deposition and attenuation of microglial activation, while others note negligible or transient effects—a consistent observation is the improvement in spatial learning and memory performance following LD-RT [259,274,275,276,277]. Mechanistically, these improvements are associated with radiation-induced modulation of neuroinflammatory pathways and transient enhancement of neurovascular coupling, which are proposed to contribute to synaptic repair and metabolic rebalancing within the hippocampal-PFC circuit.

Nevertheless, the stability and reproducibility of these cognitive benefits remain under debate. Variations in irradiation modality (photon vs. particle type), fractionation scheme, dose accumulation, and age at exposure significantly influence the outcomes. Furthermore, genetic predispositions and baseline neuroinflammatory states may modulate whether radiation acts as a mild adaptive stimulus or as a precipitating factor of cognitive decline. Hence, it would be premature to conclude that LDR universally enhances mental function. Instead, a nuanced evaluation that integrates radiation parameters, biological context, and individual susceptibility is required to distinguish adaptive neuroprotection from latent neurotoxicity.

#### 3.3.3. Remodeling and Compensation of Whole-Brain Functional Connectivity

From a systems neuroscience perspective, functional magnetic resonance imaging (fMRI) studies have provided critical evidence linking radiation exposure to alterations in large-scale brain network organization. Multiple clinical investigations have demonstrated that radiotherapy is associated with reduced functional connectivity within the DMN, a core resting-state network involved in attention, executive function, and memory. For example, Kocher et al. observed a significant post-radiotherapy decrease in DMN connectivity among patients with malignant glioma, which was accompanied by measurable declines in cognitive test performance [149]. Similarly, Mazio et al. reported comparable patterns in ALL survivors, identifying the DMN as a sensitive biomarker of radiation-induced cognitive impairment [278]. Such findings suggest that radiation may be associated with alterations in the brain’s intrinsic communication architecture, particularly in regions that sustain cognitive integration and self-referential processing.

In parallel, animal model studies have reported evidence of brain network activity under low-dose irradiation, primarily within the context of low-LET exposures. For instance, Ceyzériat et al. demonstrated in an AD rat model that fractionated low-dose brain irradiation (0.5 Gy × 5 sessions) improved memory performance without altering amyloid-β deposition, suggesting that low-dose exposure may enhance cognitive function through network-level reorganization, particularly within hippocampal–prefrontal pathways. This observation is consistent with the possibility that low-LET LDR may transiently shift neural networks toward a more energy-efficient or compensatory operating state [274].

However, findings regarding high-LET radiation (such as neutrons or heavy ions) require a distinct interpretation. Although Krishnan et al. found that chronic low-dose neutron exposure altered synaptic transmission dynamics between the hippocampus and prefrontal cortex, indicating a “rebalancing” of neural communication at the network level, this should not be interpreted simply as a beneficial adaptive response. Notably, research from the same laboratory has indicated that such neutron exposure leads to deleterious behavioral outcomes and significant CNS damage. Thus, the network “rebalancing” observed following high-LET exposure more likely represents a compensatory mechanism in response to persistent injury, rather than the functional enhancement observed in low-LET scenarios [269,279].

Collectively, the DMN and HPC–PFC circuits are suggested to be key targets of radiation-induced network plasticity. High-dose radiotherapy generally results in widespread disruption of DMN and cognitive decline, whereas low-dose irradiation may trigger limited compensatory enhancement in specific connectivity domains. Yet, systematic evidence remains scarce, and outcomes appear highly contingent on factors such as total dose, dose rate, post-exposure time window, and individual neurobiological context [280]. Future research integrating multimodal fMRI, electrophysiology, and behavioral assessments would be valuable to elucidate how LDR dynamically shapes large-scale network connectivity and to determine whether these alterations represent adaptive neuroprotection or early network instability.

### 3.4. Potential Applications of Low-Dose Radiation in Neurodegenerative Diseases

Neurodegenerative diseases constitute a highly heterogeneous group of neurological disorders and remain one of the leading causes of disability worldwide [281]. Among them, AD and PD are the most prevalent forms [282], imposing an escalating burden on global public health and socioeconomic systems. With the aging of populations, the incidence and prevalence of these disorders are projected to increase steadily in the coming decades [283]. To date, no effective therapies exist to halt or reverse their progression [284], and current treatments primarily aim to alleviate symptoms and slow clinical deterioration [285].

In recent years, adaptive responses induced by LDR have attracted growing attention as a potential neuroprotective strategy in preclinical studies of neurodegenerative diseases. Preclinical evidence suggests that LDR can trigger cellular defense mechanisms that counteract pathological processes across multiple disease models. In AD mice, low-dose irradiation has been shown to attenuate β-amyloid deposition, reduce tau hyperphosphorylation, and improve cognitive performance. These effects may be mediated by DNA damage repair, enhanced autophagic clearance, and suppression of neuroinflammatory signaling [277,286]. Notably, these favorable effects on AD-like pathology have also been observed following exposure to radiation types beyond low-dose gamma irradiation. Recent evidence indicates that exposure to mixed radiation fields (neutrons and gamma rays) can also trigger neuroprotective responses and mitigate amyloid-related deficits. Furthermore, even moderate doses of heavy ion irradiation (such as carbon ions) have been observed to reduce plaque burden and behavioral impairments in AD models, suggesting that high-LET radiation at specific doses may similarly engage adaptive or clearance mechanisms [88,287]. Similarly, in PD rat models, LDR has been reported to mitigate oxidative stress, improve mitochondrial respiratory function, and inhibit dopaminergic neuronal apoptosis, thereby alleviating radiation- or toxin-induced nigrostriatal injury [288].

At the clinical level, emerging pilot evidence suggests potential translational benefits of LDR in patients with neurodegenerative disorders. Cuttler et al. described a case of an 81-year-old woman with advanced AD, who received five low-dose head CT scans over three months, with reported improvements in cognition and daily behavior [264]. In a subsequent proof-of-concept study involving four AD patients, three demonstrated quantifiable improvements on cognitive assessment scales after LDR exposure [289]. Although limited by sample size, these preliminary findings suggest that LDR may transiently influence functional balance within degenerating neural networks.

Throughout this section, it should be noted that most evidence for adaptive or neuroprotective effects of low-dose radiation is derived from cellular and animal models. Proposed molecular mechanisms remain largely hypothetical, and any potential clinical implications should be regarded as speculative and require further experimental, translational, and clinical validation.

Despite these encouraging results, significant challenges remain before LDR can be established as a viable therapeutic intervention for neurodegenerative diseases. These challenges stem from several major issues: (1) the pathogenesis of neurodegenerative diseases remains incompletely understood, complicating mechanistic interpretation; (2) clinical studies on LDR are scarce and underpowered, often lacking rigorous control conditions; (3) substantial heterogeneity exists across experimental models, irradiation protocols, and biological endpoints, impeding cross-study comparison; and (4) dose fractionation schemes and exposure parameters vary widely, precluding the establishment of standardized mechanistic frameworks. Moreover, individual variability in radiation sensitivity and the timing of intervention (“therapeutic window”) are likely to play decisive roles in determining efficacy and safety. Future research should therefore focus on developing standardized low-dose exposure paradigms, followed by multi-model validation in transgenic animal systems and controlled clinical trials. Particular emphasis should be placed on elucidating dose–response relationships, temporal dynamics of adaptive responses, and molecular markers of neuroprotection. Such studies will be essential for defining the thresholds that separate beneficial adaptive signaling from detrimental cumulative effects, thereby providing a robust foundation for safe clinical translation.

Overall, the biological effects of LDR cannot be linearly extrapolated from high-dose responses. Instead, they likely follow a biphasic or nonlinear dose–response pattern, characterized by an intricate interplay between damage induction, repair activation, and homeostatic re-equilibration. This dynamic process—often summarized as a “damage–repair–stability” continuum—challenges the traditional LNT paradigm in radiobiology and opens new theoretical avenues for optimizing both radioprotection and therapeutic radiation use.

## 4. Conclusions and Future Directions

This review has delineated the mechanisms by which HDR impairs neurocognitive function and the adaptive responses elicited by low-dose exposure. HDR induces severe DNA damage, oxidative stress, and neuroinflammatory cascades, leading to neuronal dysfunction, suppressed neurogenesis, and failure in neural circuit reorganization, ultimately resulting in persistent cognitive decline. In contrast, LDR exhibits a dual effect, balancing potential damage with protective adaptations. Within certain thresholds, it can activate DNA repair and antioxidant defense pathways, thereby promoting neural plasticity and restoring network homeostasis. However, cumulative exposure or doses exceeding these thresholds may shift the adaptive response toward potential chronic injury.

Future research into the effects of high- and low-dose radiation on neurocognition should pursue a more systematic, targeted approach. For HDR, the focus should be on developing interventions and neuroprotective strategies during radiotherapy to minimize treatment-related cognitive impairment. This can be achieved by optimizing dose distribution and irradiation modalities, creating more efficient and less toxic radioprotective agents—such as antioxidants, anti-inflammatory drugs, and DNA repair promoters—and combining them with adjunct neuroprotective strategies. These may include targeted suppression of neuroinflammation, maintenance of BBB integrity, and promotion of neurodegeneration to improve patients’ long-term neurological outcomes.

Research on LDR, by comparison, is more exploratory and forward-looking. Future efforts should aim to dissect its dual-faced biological effects—understanding its capacity to induce mild damage while also eliciting cellular defense and neural adaptation mechanisms. There is a critical need to define the necessary dose and dose-rate thresholds at which the adaptive response transitions to potential damage, thereby delineating its biological effect boundaries. Furthermore, given emerging evidence that low-dose irradiation may improve cognitive function in neurodegenerative conditions such as Alzheimer’s disease, exploring its potential application in neuroprotection and disease intervention is of significant interest.

## Data Availability

No new data were created or analyzed in this study.
